# Thermal Conductivity of Defective Graphene Oxide: A Molecular Dynamic Study

**DOI:** 10.3390/molecules24061103

**Published:** 2019-03-20

**Authors:** Yi Yang, Jing Cao, Ning Wei, Donghui Meng, Lina Wang, Guohua Ren, Rongxin Yan, Ning Zhang

**Affiliations:** 1 College of Water Resources and Architectural Engineering, Northwest A&F University, Yangling 712100, China; yang_yi@nwafu.edu.cn; 2 State Key Laboratory of Eco-hydraulics in Northwest Arid Region of China, Xi’an University of Technology, Xi’an 710048, China; caojingxn@163.com; 3 Beijing Institute of Spacecraft Environment Engineering, Beijing 100094, China; mengdonghui@126.com (D.M.); wangxiweigood@163.com (L.W.); wqghren@126.com (G.R.)

**Keywords:** graphene oxide, thermal conductivity, vacancy defect

## Abstract

In this paper, the thermal properties of graphene oxide (GO) with vacancy defects were studied using a non-equilibrium molecular dynamics method. The results showed that the thermal conductivity of GO increases with the model length. A linear relationship of the inverse length and inverse thermal conductivity was observed. The thermal conductivity of GO decreased monotonically with an increase in the degree of oxidation. When the degree of oxidation was 10%, the thermal conductivity of GO decreased by ~90% and this was almost independent of chiral direction. The effect of vacancy defect on the thermal conductivity of GO was also considered. The size effect of thermal conductivity gradually decreases with increasing defect concentration. When the vacancy defect ratio was beyond 2%, the thermal conductivity did not show significant change with the degree of oxidation. The effect of vacancy defect on thermal conductivity is greater than that of oxide group concentration. Our results can provide effective guidance for the designed GO microstructures in thermal management and thermoelectric applications.

## 1. Introduction

Graphene oxide (GO), an oxidation product of graphene [1], has attracted much attention in recent years as a two-dimensional material [2] because of its unique mechanical and thermal properties [3,4,5]. The structure of GO is composed of oxygen functional groups connected on the base plane of a layer of carbon atoms in two-dimensional space [1]. The existence of oxygen functional groups makes its thermal transport properties quite different from those of graphene. Graphene is the best known thermal conductive material. Its thermal conductivity can reach 2000–5000 W/mK [6]. However, the oxygen functional groups on the surface of GO destroy the lattice symmetry [7] and cause local strain [8], resulting in a reduction of thermal conductivity by 2–3 orders of magnitude [9]. Nika et al. indicated that the strong phonon scattering in GO resulted in a significant decrease in thermal conductivity [10].

On the other hand, the reduction method can further regulate the concentration of oxygen functional groups, which means the thermal transport properties of GO can be regulated in a larger range. Considering the size effect, Lin and Mu calculated the effect of different degrees of oxidation on the thermal conductivity of GO [11], and revealed that the thermal conductivity converges to 8.8 W/mK [12]. In recent experiments, the thermal conductivity of GO varies from 2 to 1000 W/mK using different oxygen reduction methods [13,14,15]. GO can be used in various thermal management electronic devices [16], such as electronic cooling [17], thermal diodes [18] and thermal logic circuits [19] due to the ability to adjust thermal conductivity. In addition, GO also shows good thermoelectric properties [4,20]. Therefore, considering the potential applications of GO in thermal management and thermoelectric energy conversion, it is necessary to study the thermal conductivity of GO.

In the process of preparation and reduction of GO, structural damage and vacancy defect are inevitable. GO is often regarded as a monolayer graphene with both oxygen functional groups and vacancy defects [1]. Renteria et al. revealed that the thermal conductivity of GO films is anisotropic [21]. In recent years, some progress has been made in the study of GO thermal conductivity. Zhao considered the effect of various defects on thermal conductivity of GO strips with fixed length [22]. The thermal conductivity of materials depends on phonons, and the phonon scattering is enhanced by GO defects, thus reducing the thermal conductivity [15,23]. On the other hand, with the presence of oxygen functional groups and doping defects, the thermal conductivity may be further reduced [9]. However, current studies cannot accurately describe the coupling effect of degree of oxidation and vacancy defects on thermal conductivity. Quantitative analysis of this problem is necessary.

In this study, the thermal conductivity of GO is calculated based on the non-equilibrium molecular dynamics method. Considering the coupling effect of oxygen group concentration and the ratio of vacancy defects, the variation of in-plane thermal conductivity of monolayer GO is studied, and the empirical formula for the ratio of vacancy defect, degree of oxidation and thermal conductivity of GO is established.

## 2. Model and Methodology

GO has two main surface groups, hydroxyl and epoxy groups [24]. The main factor affecting the thermal conductivity of GO is the content of functional groups (degree of oxidation) rather than the type of functional groups [22]. Therefore, only one functional group type of hydroxyl (-OH) is considered in this work.

**Figure 1:**
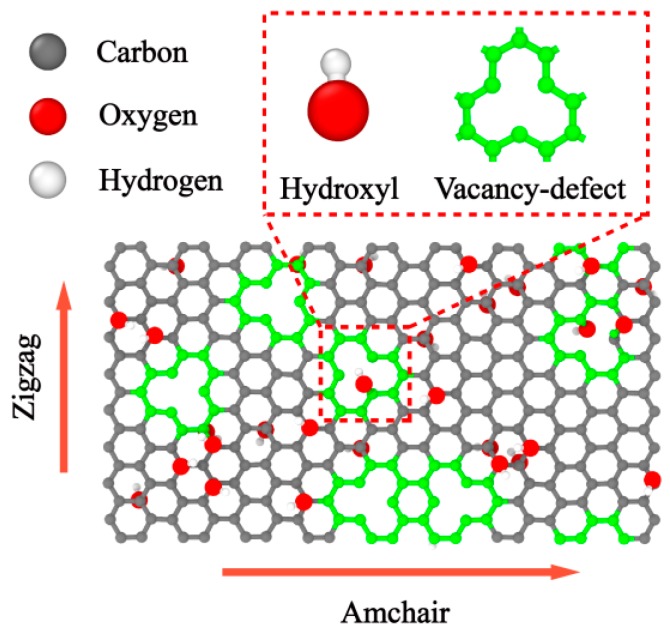
Schematic picture of graphene oxide (GO) with randomly distributed vacancy defects and hydroxyl groups.

Here, GO with randomly distributed vacancy defects and hydroxyl groups was built as shown in Figure 1. To make the calculation model more consistent with the actual situation, the quenching process of GO was simulated using the ReaxFF reactive force field under NPT ensemble [25,26]. The GO, established with several different initial functional group concentrations, was first gradually heated from 300 to 500 K over a time span of 0.2 ns, then annealed at 500 K for 0.2 ns, and subsequently quenched to 300 K over a time span of 0.2 ns. Finally, the model was further annealed at 300 K and zero pressure for the duration of 0.2 ns to ensure complete equilibration of the structure. Thus, each GO model was obtained with the final functional group concentration after quenching.

Through the above steps, the hydroxyl groups and vacancy at several different ratios were introduced in the model. The hydroxyl groups were randomly attached to the carbon atoms on both sides of the graphene basal plane at different degrees of oxidation ranging from 0% to 10%, while removing the carbon atoms from the GO sheet on the surface defect from 0% to 2%.

**Figure 2:**
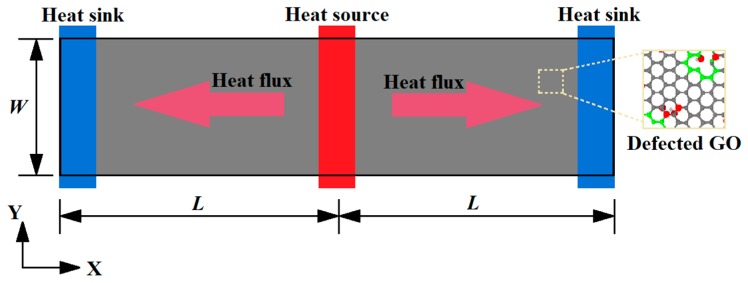
Schematic model for thermal conductance of GO using periodic boundary conditions.

In the present study, the dynamic response of the system shown in Figure 2 was revealed by a molecular dynamics (MD) approach. The MD simulations were carried out by using the large-scale atomic/molecular massively parallel simulator (LAMMPS) [27]. The all-atom optimized potential for liquid simulations (OPLS-AA) was used for the study of GO thermal conductivity to improve the computation efficiency [28,29,30].

To avoid the computational problems created by high frequency vibration caused by bond stretching energy (-OH) and bond angle bending energy (C-O-H), the SHAKE algorithm was adopted to fix atoms. Coulomb interactions were computed by using the particle-particle particle-mesh (PPPM) algorithm [31]. In this work, the thermal conductivity was computed by reverse non-equilibrium molecular dynamics (RNEMD) simulations in a microcanonical NVE ensemble [32]. The key point of the method is to impose a heat flux through the system and to determine the temperature gradient and temperature junctions as a consequence of the imposed flux. The fastest descent method was used to redistribute the atomic positions.

The above systems were equally divided into 100 thin slabs along the heat transfer direction, with the heat source and sink each taking one of the slabs. The heat source (hot slab) and sink (cold slab) slabs were located at the middle and the two ends of the model, respectively. The periodic boundary conditions were applied in the X and Y direction. A time step of 0.1 fs was selected for integration of the equations of atomic motion in the simulations. The system reached the equilibrium state at 300 K in Nosé-Hoover thermal bath for 0.2 ns. Then, the system was switch linear fitted to the NVE ensemble to exchange the kinetic energies (every 1000-time steps) between the coldest atom in the heat sink slab and the hottest atom in the heat source slab for 0.8 ns. The total heat flux J can be obtained from the amount of the injected/released two slabs by exchanging the kinetic energies Equation (1).

(1) $$J=\frac{\sum_{N_{\mathrm{tranfers}}}\frac{1}{2}(mv_{h}{}^{2}-mv_{c}{}^{2})}{t_{\mathrm{transfer}}},$$

where $N_{\mathrm{tranfers}}$ is the total number of exchanging the kinetic energies, $t_{\mathrm{tranfers}}$ is the time over which the exchanging simulation is started, *m* represents the mass of the atoms, $v_{h}$ and $v_{c}$ are the velocities of the hottest atom of the cold slab and the coldest atom of the hot slab, respectively. When the heat flow in the structure reaches the non-equilibrium steady state, the temperature profiles is collected to obtain the temperature gradient as Equation (2).

(2) $$T_{i}=\frac{2}{3Nk_{B}}\sum_{j}\frac{p_{j}^{2}}{2m_{j}},$$

where $T_{i}$ is the temperature of the N number atoms in *i* -th slab. $m_{j}$, $v_{j}$ and $p_{j}$ represent the mass and velocity and momentum of the atom *j* in *i* -th slab, respectively. The term $k_{B}$ is Boltzmann’s constant. The temperature profiles are obtained by averaging results of the last 8 million timesteps.

**Figure 3:**
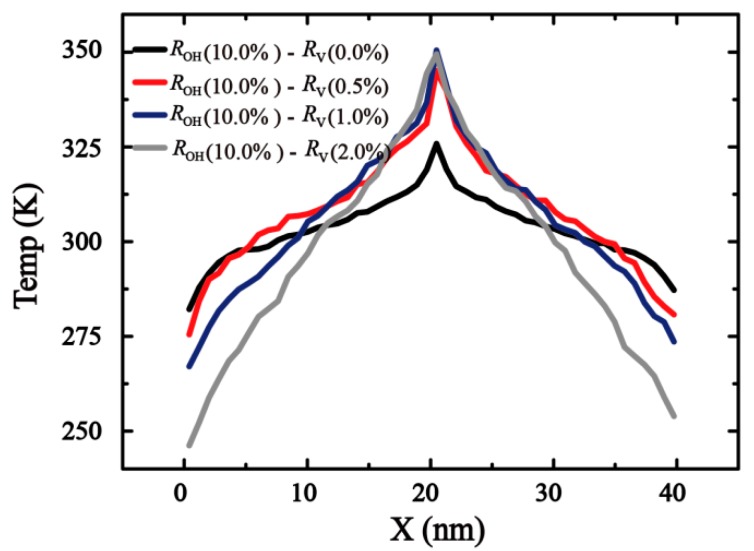
Schematic plot for reverse non-equilibrium molecular dynamics (RNEMD) simulations and equilibrium temperature profiles for GO.

Four typical samples of the temperature profiles of monolayer GO are shown in Figure 3, where the temperature gradient ∇T (dT/dx) was obtained by linear fitting in the linear region of the profile along the longitudinal heat flux direction in Figure 3. The thermal conductivity $\kappa_{G}$ can be calculated as Equation (3).

(3) $$\kappa_{G}=\frac{J}{2A\nabla T},$$

where A is the cross-section area of corresponding models and the constant 2 in the denominator accounts for the fact that the system is periodic.

## 3. Result and Discussion

**Figure 4:**
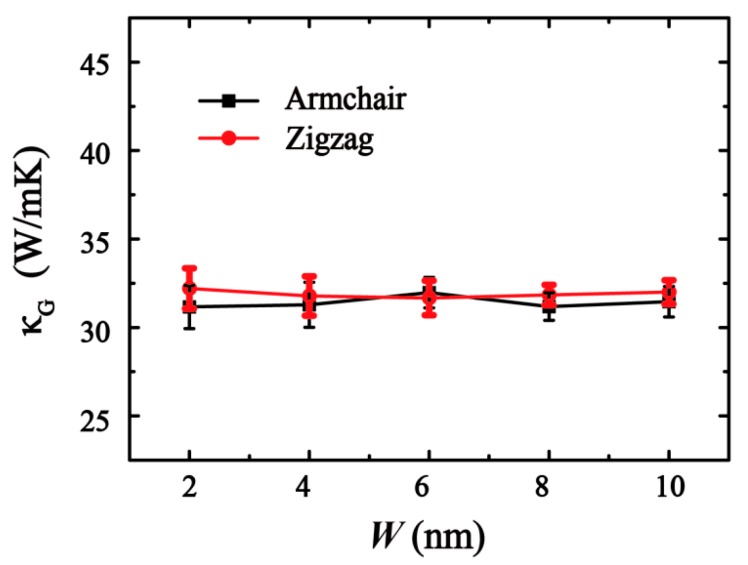
Curve of thermal conductivity with different sample width. The width varies in [0, 10] nm.

First, the effects of the sample width on the thermal conductivity was investigated through MD simulations. As shown in Figure 4, in the range of 2 to 10 nm for different chirality with a fixed length of 20 nm, the measure of increasing width *W* acquired a convergent thermal conductivity.

**Figure 5:**
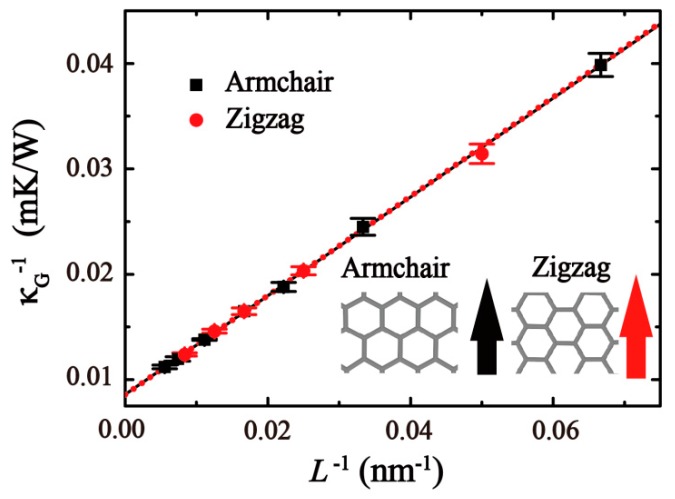
The relationship of length and thermal conductivity in GO ($R_{\mathrm{OH}}$: ~10%) along zigzag (red) and armchair (black) directions at 300 K.

Then, the effects of the sample length on the thermal conductivity ($\kappa_{G}$) along the zigzag and armchair directions were explored with the length varying from 20 to 180 nm and a fixed width of 2 nm. The results (see Figure 5) clearly show that the thermal conductivity does not depend on the sample’s width. A linear relationship of the inverse length and inverse thermal conductivity can be observed. This means that the thermal conductivity increases with the length, two fitting functions are $\kappa_{G(\mathrm{Zigzag})}^{-1}=0.4704L^{-1}+0.00857$ and $\kappa_{G(\mathrm{Armchair})}^{-1}=0.4697L^{-1}+0.00856$.

The relationship between $\kappa_{G}^{-1}$ and $L_{}^{-1}$ can also be expressed as [33]:

(4) $$\kappa_{G}^{-1}=\kappa_{\infty }^{-1}(\frac{2l}{L}+1),$$

where *l* is the mean free path (MFP) of phonon. $\kappa_{\infty }$ denotes the thermal conductivity in infinite length. Through Equation (4), the thermal conductivity $\kappa_{\infty }$ along the zigzag and armchair directions was found to be 116.82 and 116.68 W/mK, respectively. The corresponding MFP of phonon values *l* were 27.45 nm (along zigzag direction) and 27.44 nm (along armchair direction), which are much smaller than that of graphene (~775 nm) [6].

Through the classical lattice heat transport theory, the thermal conductivity of low-dimensional material can be calculated by κ=Cvl, where C is the specific heat, v is the group velocity. Previous literature has indicated that the values of C and v changed little by analyzing phonon density of states in GO [15]. This explains why the thermal conductivity of GO is smaller than that of graphene.

To study the coupling effects of the hydroxyl-group and vacancy defects on the thermal conductivity of GO, we defined a ratio between oxygen and carbon atoms $R_{\mathrm{OH}}$ to describe the degree of oxidation. Also, $R_{V}$ is defined as the ratio of vacancy defect in the system, which can be calculated by the density of atoms removed from the pristine GO.

**Figure 6:**
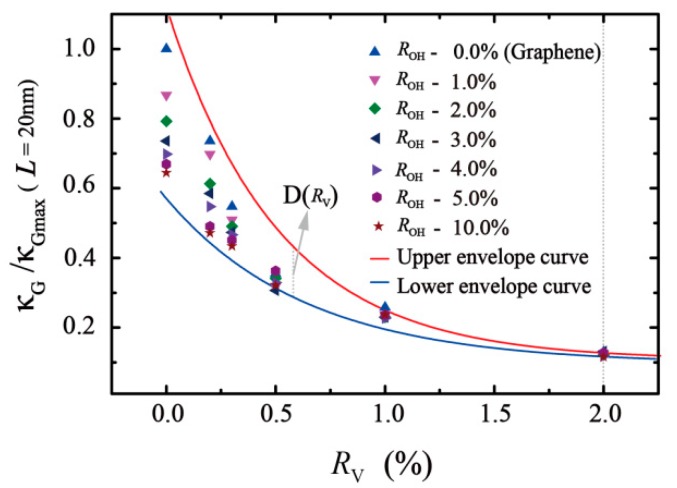
The relative thermal conductivity of GO with varying degrees of oxidation and vacancy defect ratio in the same sample size of 20 nm. Six different symbols indicate the different degree of oxidation with varied vacancy defect ratios, the red and blue line denote the fitting curves.

From Figure 6, the concentration of functional groups and the ratio of vacancy defects have a negative impact on the thermal conductivity of the structure in a certain degree. For a known concentration of functional groups, the thermal conductivity of the structure decreases gradually with the increase in vacancy defects in the structure. The decline in thermal conductivity is no longer obvious with the increase in vacancy defect ratio. When the vacancy defect $R_{V}\leq 1.0\%$, the thermal conductivity is very sensitive to both the change in vacancy defect and the concentration of functional groups. For GO without vacancy defect, the thermal conductivity drops most significantly while the functional group concentration increases. When $R_{V}$ exceeds 2.0%, the functional group concentration has little effect on thermal conductivity.

According to the results, the lower and upper envelope curves of nonlinear fitting are drawn in Figure 6. The upper curve in red indicates the thermal conductivity of the model is only affected by the vacancy defect ratio. The fitting formula is $\kappa_{G}/\kappa_{G\max}=0.1142+0.8859e^{-R_{V}/0.5126}$. The lower envelope curve in blue is the thermal conductivity of the system with 10% oxidation affected by the vacancy defect ratio. The fitting formula is $\kappa_{G}/\kappa_{G\max}=0.1003+0.5034e^{-R_{V}/0.6365}$. The region between the lower and upper envelope curve indicates all the cases of coupling effects between a single vacancy ($R_{V}$: 0 ~ 2%) and the hydroxyl group ($R_{\mathrm{OH}}$: 0 ~ 10%) in 20 nm length (Figure 6). The simulation results also reveal that the effect of vacancy defects on thermal conductivity of GO is greater than that of functional group concentration.

To explore the coupling effect of such factors, we define the $D(R_{V})$ (see Figure 6) as the difference between the upper envelope curve and lower envelope curve at a same ratio of vacancy. $D(R_{V})$ decreases as $R_{V}$ increases and approximately approaches zero when $R_{V}>2.0\%$. Results indicated that the vacancy has a strong effect on thermal conductivity compared with the oxygen functional concentration. For example, when $R_{V}=2.0\%$, the thermal conductivity with samples size of 20 nm is about 6.01 W/mK, regardless of the changing concentration of the functional group.

**Figure 7:**
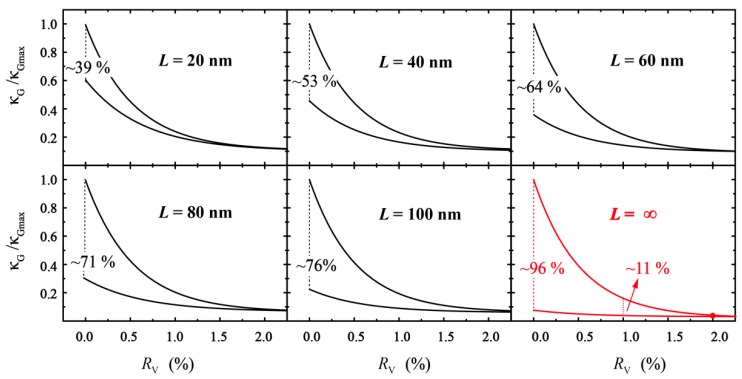
Relative thermal conductivity in different sample sizes.

To further investigate the thermal conductivity on a macroscopic scale, the coupling effect of $R_{\mathrm{OH}}$ and $R_{V}$ with five different GO lengths was employed. The ranges of the GO envelope are shown in black curves in Figure 7. As the length (*L*) of the GO sheet increases, the area between the lower and upper envelope curves is extended.

Combined with Equation (4), the thermal conductivity is extrapolated to infinite size. As the red curves show in Figure 7, the upper envelope indicates that the thermal conductivity of graphene tends to converge with the increase in the defect ratio. The results are similar to those obtained by Malekpour [34,35]. Also, the lower envelope is a thermal conductivity of *R*_OH_ = 10% GO. Two lines indicate that the maximum range of thermal conductivity can be up to 96%. With the increase of $R_{V}$, the regulatory range of functional groups decreases gradually. The range of functional group regulation is only ~11% when the vacancy defect ratio is at 1%. When the vacancy defect reaches 2%, the concentration of functional groups has little effect on the thermal conductivity. Therefore, in order to obtain a larger range of thermal conductivity control capabilities, it is necessary to reduce the vacancy defects in GO.

**Figure 8:**
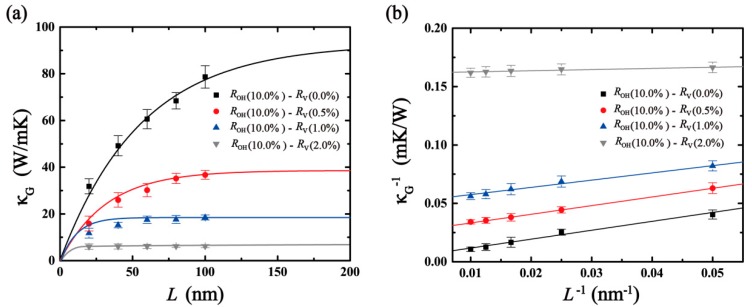
Length dependence of defect-GO’s thermal conductivity. Solid lines are best fit to Equation (4). (**a**) The relationship between κ and *L*, (**b**) the relationship between κ^−1^ and *L*^−1^.

As shown in Figure 8, with the increase in vacancy defects in GO, the size effect is no longer obvious. The thermal conductivity converges to 6.23 W/mK with a 2% defect. This proves that the thermal conductivity of defect-GO is less dependent on model length than that of the corresponding graphene and GO, since the thermal conductivity of defect-GO is mainly influenced by short-range acoustic and optical phonons which are length-independent [36]. Also, the less length-dependent thermal conductivity of defect-GO indicates that the long-range acoustic phonons are mainly scattered at vacancy. Moreover, a linear relationship of the inverse length and inverse thermal conductivity can be observed in the four types of defect ratio (see Figure 8b). Through formula (4), the corresponding MFPs of phonon are shown in Table 1. When the simulated size is larger than the MFP of phonon, the ballistic transport no longer plays a leading role and the thermal conductivity gradually converges [10]. Therefore, the larger the defect ratio, the smaller the simulation domain size as the GO thermal conductivity converges.

**Figure 9:**
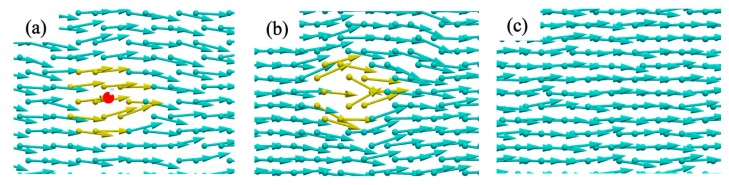
Spatial distribution of heat flux by vector arrows on each atom under non-equilibrium steady state. (**a**) A hydroxyl group, (**b**) one single vacancy, (**c**) graphene.

To elucidate the mechanism of heat transfer of GO sheets, the spatial distribution of the heat flux by vector arrows on each atom under non-equilibrium steady state is shown in Figure 9, which displays the heat flux of GO for the specified structure.

The atomic heat flux is defined from the expression: $\vec{J_{i}}=e_{i}\vec{v_{i}}-S_{i}\vec{v_{i}}$, where $e_{i}$*,* $v_{i}$, and $S_{i}$ are the energy, velocity vector and stress tensor of each atom *i*, respectively [37]. It can be obtained by calculating the atomic heat flux in the MD simulations and the results are averaged over 1 ns. The vector arrows show the migration of the heat flux on the GO and vividly reflect the transformation of the heat flux path as well as the phonon scattering around the vacancy/hydroxyl group regions.

The heat flow scattering occurs at the vacancy and hydroxyl group regions on the surface of GO (see Figure 9). When a propagating heat flux tries to pass through a barrier in GO, under a single vacancy defect, the heat flow not only diffuses out of the plane, but also disturbs the heat flow around the pore in the plane. The heat flow shows irregular transmission while the addition of functional groups only slightly disturbs the surrounding heat flow. In other words, the hydroxyl groups do not break the underlying hexagonal lattice and preserve relatively well the lattice symmetry of carbon atoms and integrity, thus disturb the thermal transport weakly. In contrast, the presence of vacancies reduces the thermal conductivity of graphene significantly as they break the in-plane network of *sp*^2^ carbon bonds. Therefore, among the factors affecting thermal conductivity, the scattering effect of functional groups is less than that of vacancy defects. As shown in the previous analysis, when the vacancy defect ratio reaches a certain value, the perturbation caused by functional groups is covered by vacancy defects and the influence is negligible, thus, the change in thermal conductivity with the concentration of functional groups is no longer obvious.

## 4. Conclusions

In summary, classical MD simulations were performed to investigate the thermal conductivity of GO with vacancy defect. Based on the simulation results, we found that GO has a significant size effect. The size effect of GO deteriorates with the increase in vacancy defects. It was also found that the effect of vacancy defects on thermal conductivity is more obvious than the degree of oxidation. With the increase in vacancy defects, the ability of functional group concentration to regulate the thermal conductivity of GO decreases. When the vacancy defect ratio is over 2%, the thermal conductivity does not show significant change with the degree of oxidation. This study provides theoretical guidance for the design and manufacture of thermoelectric and thermal management devices using GO as a raw material.

## Figures and Tables

**Table 1:** The mean free path (MFP) of phonon for four types of defect ratio in GO.

Type	Fitting Functions	MFP of Phonon
$R_{\mathrm{OH}}(10\%)-R_{V}(0.0\%)$	$\kappa_{G}^{-1}=0.4697L^{-1}+0.00856$	27.44 nm
$R_{\mathrm{OH}}(10\%)-R_{V}(0.5\%)$	$\kappa_{G}^{-1}=0.4422L^{-1}+0.02581$	8.57 nm
$R_{\mathrm{OH}}(10\%)-R_{V}(1.0\%)$	$\kappa_{G}^{-1}=0.2199L^{-1}+0.05130$	2.14 nm
$R_{\mathrm{OH}}(10\%)-R_{V}(2.0\%)$	$\kappa_{G}^{-1}=0.0895L^{-1}+0.15162$	0.29 nm
